# Informational and Neuromuscular Contributions to Anchoring in Rhythmic Wrist Cycling

**DOI:** 10.1007/s10439-012-0680-7

**Published:** 2012-10-26

**Authors:** Melvyn Roerdink, Arne Ridderikhoff, C. E. Peper, Peter J. Beek

**Affiliations:** MOVE Research Institute Amsterdam, Faculty of Human Movement Sciences, VU University Amsterdam, Van der Boechorststraat 9, 1081 BT Amsterdam, The Netherlands

**Keywords:** Motor control, Anchoring, Coordination dynamics, Trajectory variability, Auditory-motor synchronization, Wrist posture, Electromyography

## Abstract

Continuous rhythmic movements are often geared toward particular points in the movement cycle, as evidenced by a local reduction in trajectory variability. These so-called anchor points provide a window into motor control, since changes in the degree of anchoring may reveal how informational and/or neuromuscular properties are exploited in the organization of rhythmic movements. The present experiment examined the relative contributions of informational timing (metronome beeps) and neuromuscular (wrist postures) constraints on anchoring by systematically varying both factors at movement reversal points. To this end, participants cycled their right wrist in a flexed, neutral, or extended posture, either self-paced or synchronized to a metronome pacing peak flexion, peak extension, or both peak flexion and extension. The effects of these manipulations were assessed in terms of kinematics, auditory-motor coordination, and muscle activity. The degree of anchoring seen at the reversal points depended on the degree of compatibility of the prevailing configuration of neuromuscular and informational timing constraints, which had largely independent effects. We further observed systematic changes in muscular activity, which revealed distinct contributions of posture- and muscle-dependent neuromuscular properties to motor control. These findings indicate that the anchor-based discretization of the control of continuous rhythmic wrist movements is determined by both informational timing and neuromuscular constraints in a task-specific manner with subtle interactions between the two, and exemplify how movement variability may be exploited to gain such insights.

## Introduction

Since the seminal work of Bernstein^5^ it has been duly recognized in the motor control literature that variability is a cardinal feature of biological movement. Even if we repeat the same task over and over again, no movement trajectory is identical to another. An instance of repetition without repetition, as Bernstein described it so aptly.

A critical decision for researchers of human movement is how to deal with this variability in the analysis of movement data, how to account for it theoretically, and how to exploit motor variability as a window into motor control. Various approaches in this regard become readily apparent when taking a bird’s eye view on the motor control literature of the past four decades or so. A case in point is the time-keeper approach in which models of motor timing, consisting of a noisy clock (or clocks) that times (or time) the execution of movement with a noisy motor delay, are derived and tested on the basis of the statistical properties of inter-tap intervals.^19^,^38^ The alternative dynamical systems approach focuses not only on the timing of motor events but also on the local and global dynamical properties of the movement trajectories themselves,^1^,^4^,^14^,^17^,^24^,^35^,^36^ be it on the basis of an explicit model,^17^ or through application of non-linear time-series analyses to assess task-dependent changes in the flow strength and curvature of the vector fields of the phase portraits of the rhythmic movements in question.^36^ Interestingly, studies from both approaches have indicated that rhythmic movements are often not controlled continuously over the entire movement cycle but may take a discrete form with the movements being consistently steered to particular points or regions in the movement cycle,^3^,^6^,^7^,^14^,^18^,^23^,^27^,^28^,^30^–^33^,^39^ as was in fact already observed a long time ago by Wachholder and Altenburger.^37^ To anticipate, this “anchoring” phenomenon^3^,^6^,^7^,^14^,^23^,^30^–^33^ was exploited in the present experimental study to probe and tease apart the contributions of informational timing (i.e., metronome beeps) and neuromuscular (i.e., wrist postures) constraints to motor control.

In his initial study of juggling, Beek^3^ observed that the cyclical hand movements and ball motions appeared to be organized around certain spatial locations (e.g., the throws and the zeniths) as evidenced by a marked reduction in trajectory variability at these locations compared to other locations. He hypothesized that these locations of reduced variability served as intentional attractors or organizing centers within and for the movement cycle and therefore dubbed them “anchor points.” According to this hypothesis, anchor points may reflect locations in the perceptual-motor workspace where task-specific information is available, for instance about the required timing when rhythmic limb movements are coordinated with an external signal or event. However, later experiments^6^,^9^,^32^ showed that anchor points may also reflect points or regions in the perceptual-motor workspace where functional, task-related neuromuscular properties are exploited, such as the ability to store and release energy.^16^ Indeed, with the wrist in extreme positions, wrist oscillations involve only activation of shortened agonist muscles, resulting in reduced reversal point variability accompanied by a shorter movement duration in the direction of the anchor point^32^ and suggesting that the rebound results from passive moments of elongated antagonist muscles.^9^,^12^,^37^

Informational timing and neuromuscular constraints may in fact be operative at the same location. In self-paced rhythmic forearm rotations, for example, peak pronation was characterized by lower variability than peak supination,^6^ probably due to inherent neuromuscular differences between the contributing muscle groups. Interestingly, acoustically-paced bimanual in- and antiphase rhythmic movements were most stable when peak pronation rather than peak supination was time-locked with the pacing signal,^6^,^9^ i.e., when the metronome beeps coincided with the anchor point location identified in unpaced forearm rotations. Thus, informational timing and neuromuscular constraints on anchoring may coincide, which raises the need to determine their potential interplay and relative contributions to anchoring in the execution of continuous rhythmic movements.

Although both acoustic pacing^1^,^6^,^14^,^20^ and wrist posture^32^,^33^ are known to induce anchor points, as evidenced by reduced reversal point variability and shorter movement duration in the direction of this point,^32^ their combined effects have not been studied to date in a systematic and well-controlled manner. In the current experiment, a pacing signal provided an informational constraint on anchoring at peak flexion and extension in flex-on-the-beat and extend-on-the-beat conditions, respectively (*I* in Table 1). This informational timing constraint was absent in conditions without pacing and balanced over the two reversal points in double pacing (i.e., flex-and-extend-on-the-beat) conditions (*i* in Table 1). Likewise, we used flexed or extended wrist postures to impose neuromuscular constraints on anchoring at peak flexion or extension, respectively (*M* in Table 1), which were balanced over reversal points in the neutral posture (*m* in Table 1).

**Table 1:** Schematic overview of experimental conditions and experimentally induced informational timing (*I*) and neuromuscular (*M*) constraints on anchoring at the flexion or extension endpoints

	No pacing		Flex-on-beat		Extend-on-beat		Double pacing	
	Flexion	Extension	Flexion	Extension	Flexion	Extension	Flexion	Extension
Flexed	*M*		*M*		*M*		*M*	
			*I*			*I*	*i*	*i*
Neutral	*m*	*m*	*m*	*m*	*m*	*m*	*m*	*m*
			*I*			*I*	*i*	*i*
Extended		*M*		*M*		*M*		*M*
			*I*			*I*	*i*	*i*

Lower case letters are used if those constraints were balanced over endpoints

Specific expectations were derived from the general assumption^6^ and empirical indications^32^ that informational timing and neuromuscular constraints on anchoring are independent and, hence, have additive effects. Thus, we expected anchoring to occur on peak flexion if *M* and *I* coincide here (*viz*. flex-on-the-beat condition with the wrist flexed), and likewise on peak extension for the extend-on-the-beat condition with the wrist extended. In the double pacing condition with the wrist flexed or extended, we expected anchoring on peak flexion or peak extension, respectively, due to *M* in combination with the informational timing constraint balanced over endpoints (*i*). Flex-on-the-beat and extend-on-the-beat with the neutral wrist (*I* added to balanced *m*) were expected to induce anchoring on peak flexion and peak extension, respectively. The conditions in which informational timing and neuromuscular anchor points were in conflict (*I* and *M* at opposite endpoints) or in balance (neutral posture with no pacing or double pacing) were crucial in determining their relative contribution to the control of continuous rhythmic movements.

Although, to our knowledge, analyses of anchoring have thus far been limited to the level of kinematics, there are clear indications that timing rhythmic wrist movements to a specific point in the movement cycle involves systematic changes in muscle activity.^12^,^37^ For this reason, we also assessed the muscular activity associated with anchoring. In particular, we were inspired by Wachholder and Altenburger’s^37^ demonstration that voluntary emphasis on the flexion or extension phase of rhythmic movements involved shorter movements in the accentuated direction which begun with a discontinuity at or near the non-accentuated reversal point (see also Balasubramaniam *et al*.^1^). These kinematic signs of accentuation were accompanied by changes in flexor and extensor muscular activity: a longer period of stronger activity was observed in the accentuated direction, followed by a relatively long pause prior to the onset of muscular activity corresponding to the non-accentuated direction (p. 632 and Fig. 6 on p. 635).^37^ These early observations suggest that accentuating or emphasizing movements to a particular point in the movement cycle, in line with the notion of anchoring, are brought about by changes in the duration, timing, and amplitude of muscular activity. Hence, we expected that the muscle(s) instantiating movement in the anchored direction would show increased activity and longer burst duration, in combination with modifications in the relative timing between flexor and extensor bursts.

## Materials and Methods

### Participants

Six males and seven females (aged 19–29 years) volunteered to participate in the study. All were right-handed according to their scores on a shortened version of the Edinburgh handedness inventory^26^ (mean laterality quotient: 79.0%). Participants gave their written informed consent prior to the experiment, which was approved by the local ethics committee.

### Apparatus

Participants were seated in a height-adjustable chair with their right forearm resting on a tabletop with adjustable supports to prevent forearm movement and to secure its neutral position (i.e., centered between pronation and supination extremes). Only flexion and extension movements about the wrist were allowed. The right hand was strapped against a flat, vertically oriented manipulandum mounted on a potentiometer whose axis was aligned with the flexion–extension axis of the wrist. Surface electromyograms (EMG) were obtained from m. flexor carpi radialis (FCR) and m. extensor carpi radialis (ECR). After cleansing and abrasion of the skin, disposable electrodes were positioned in the center of the muscle belly on the line from origin to insertion in a bipolar arrangement with a center-to-center distance of 2 cm. Computer-generated acoustic pacing signals (50 ms beeps, pitch: 440 Hz) were presented through a speaker positioned in front of the participants. Wrist angular position, EMG signals, and acoustic pacing signals were synchronously sampled at 1000 Hz. During stationary wrist posture and practice trials (see below), a feedback display was positioned at a distance of about 2 m at “2 o’clock” in front of the participant providing concurrent visual feedback of wrist angular position. The display consisted of a semicircular bow comprising a continuous array of 448 light-emitting diodes (LEDs) that represented wrist angular positions over a range of 150°. During experimental trials online feedback was visible for the experimenter only, because during those trials the feedback display was rotated towards the experimenter and participants were instructed to direct their gaze at a smiley positioned at eye-height 2 m in front of them (at 12 o’clock). A cover prevented vision of the moving hand.

### Procedure

Prior to the experiment proper, participants were instructed to synchronize smooth oscillatory movements about the right wrist with a 3-Hz metronome, with both peak flexion and peak extension coinciding with a beep (i.e., movement frequency: 1.5 Hz). Only participants that were able to stably perform this double pacing condition within three practice trials were included in the experiment (one candidate participant failed to meet this criterion). Next, the EMG electrodes were applied and the remaining 12 participants performed maximum voluntary contractions (MVC) by generating twice a maximal isometric flexion or extension torque about the right wrist for 3 s.

The experiment examined wrist cycling in three wrist postures (i.e., flexed, neutral, and extended) crossed with four acoustic pacing conditions (i.e., no pacing, flex-on-the-beat, extend-on-the-beat, and double pacing). Participants performed all 12 conditions six times, resulting in 72 trials per participant. Trials were presented in blocks with the three wrist postures providing the first level of blocking (3 × 24 trials) and acoustic pacing (4 × 6 trials) the next. The first trial was always a practice trial in which participants received concurrent visual feedback of their wrist angular position and movement amplitude. In the next five trials, the LED feedback display was rotated towards the experimenter and participants were instructed to direct their gaze to the smiley in front of them to prevent potential gaze anchoring effects.^31^,^32^ The order of the wrist posture blocks was counterbalanced over the 12 participants. The acoustic pacing blocks within the wrist posture blocks were presented in (semi-)random order with the restriction that the experiment never started with the no pacing condition (see below).

At the start of each wrist posture block, participants were positioned comfortably in the apparatus and the range of wrist motion was determined. Participants adopted their maximal flexion and extension position, each for about 5 s. The center in between these extremes was taken as the neutral position. Subsequently, participants held their wrist at nine different angular positions which were administered in random order (stationary trials: −60°, −45°, −30°, −15°, 0°, 15°, 30°, 45°, and 60°, with 0° corresponding to the individually determined neutral wrist position) to estimate the muscular effort necessary for maintaining these positions against the forces generated by joint stiffness. These experimentally induced stationary wrist orientations all fell well within the individually determined maximal range of motion (155.0 ± 13.5°). Participants had to maintain the LED feedback signal at the designated position (indicated by a marker on the feedback display; tolerance range: ±2.5°) for 10 s, while wrist angular position and EMG were recorded.

Subsequently, one of the three wrist posture blocks was performed. Participants oscillated their wrist with 15° amplitude (range 30°) around −45°, 0°, and 45° in flexed, neutral, and extended blocks, respectively. They were instructed to cycle as smoothly as possible and to synchronize peak flexion (extension) to the beat of the metronome in the flex(extend)-on-the-beat condition. In these single pacing conditions the metronome frequency was 1.5 Hz. In the double pacing condition the metronome was set at 3.0 Hz and flexion and extension excursions were synchronized to consecutive beeps, resulting in a movement frequency of 1.5 Hz as well. In the no pacing condition, participants were instructed to cycle their hand as smoothly as possible at about the same rate as in the paced trials. All trials lasted 30 s (i.e., 45 cycles for pacing trials). To facilitate trial initiation, the experimenter guided the hand to the flexion excursion position in the flex-on-the-beat condition (i.e., −60°, −15°, and 30° for flexed, neutral, and extended wrist posture blocks, respectively) and to the extension excursion position in the extend-on-the-beat condition (i.e., −30°, 15°, and 60°, respectively). In the no pacing and double pacing conditions, the hand was guided to the required center region (i.e., −45°, 0°, or 45°). Trials were repeated if the mean amplitude range deviated more than 10° from the required range, if mean wrist posture during the trial deviated more than 10° from the prescribed mean wrist posture (i.e., −45°, 0°, or 45°), or if mean movement frequency deviated more than 0.01 Hz from the prescribed frequency in the pacing trials (leading to drift in the phase relation between hand excursions and metronome beats) while for the no pacing condition mean movement frequencies lower than 1.4 Hz or higher than 1.6 Hz were penalized. Each wrist posture block lasted approximately 25 min after which a break of at least 5 min was introduced. The experiment lasted 2–2.5 h, including breaks.

### Data Analysis MVC and Stationary Trials

FCR and ECR recordings were band-pass filtered (10–400 Hz, second-order bi-directional Butterworth filter) and subsequently whitened using a fifth-order autoregressive filter.^34^ The highest root mean square (RMS) value in 250 ms windows in the two MVC attempts was defined as the MVC value and used for normalization. For each stationary position trial, the average RMS value (normalized to MVC) over the last 7 s was used as a measure for the muscular effort to maintain that specific position.

### Data Analysis of Experimental Trials

#### Preprocessing and Trial Selection

We had to exclude 11 trials from further analysis due to data collection errors, as well as 16 unpaced trials that did not meet the abovementioned frequency criterion. Potentiometer data (hand movement) of the remaining trials were low-pass filtered using a second-order bi-directional Butterworth filter (cut-off frequency: 15 Hz). The first five cycles of each trial were excluded to eliminate possible transient effects. For the acoustically paced trials, the phase *ψ* (in °) relative to the metronome was determined for each cycle as *ψ*_*i*_ = 360°·(*t*_*y*,*i*_ − *t*_*x*,*i*_)/(*t*_*x*,*i*+1_ − *t*_*x*,*i*_), where *t*_*y*,*i*_ indicates the time of the *i*th peak flexion (extension) excursion and *t*_*x*,*i*_ corresponds to the moment of the *i*th metronome beep that specified peak flexion (extension) excursion.^29^ Positive values of *ψ* implied that the hand (*y*) was lagging the metronome (*x*). Mean and standard deviation of *ψ* (i.e., $\bar{\psi }$ and σ_*ψ*_) were determined using circular statistics.^22^ For each trial, a segment of 20 cycles was selected showing 1:1 frequency synchronization (i.e., no drift in *ψ*) with the required phase relation between hand excursions and metronome beeps, identified using the following criteria: (a) mean movement frequency *f* (inverse of mean period between consecutive peak extensions) between 1.49 and 1.51 Hz, (b) $\bar{\psi }$ between −90° and 90° and (c) σ_*ψ*_ smaller than 27°. These criteria were violated in 13 trials, which were excluded from further analysis. For the remaining 680 trials (94.4% of all trials) dependent variables regarding task performance, local and global kinematics, and EMG were determined.

#### Task Performance

Task performance was evaluated in terms of mean movement frequency *f*, mean amplitude *A*_*θ*_ of wrist angular position time series *θ*, and the deviation from the required mean wrist position Δ*θ*_req_ (*viz*. difference between the required center of oscillation and the actual center in between the movement reversal points; for negative (positive) Δ*θ*_req_ the wrist was on average more flexed (extended) than required).

#### Local and Global Kinematics

Spatial variability of wrist flexion and extension reversals was expressed by the respective standard deviations of positional minima and maxima of the potentiometer data (σ_spatial_ in °). Apart from these local kinematic characteristics, global properties of the wrist oscillations were assessed using phase portraits (i.e., wrist angular velocity $\dot{\theta }$ as a function of wrist angular position *θ*) and Hooke’s portraits (i.e., wrist angular acceleration $\ddot{\theta }$ as a function of *θ*). To this end, *θ* was mean centered and normalized to unit amplitude (i.e., −1 and +1 imply mean peak flexion and mean peak extension excursions, respectively). The movement duration of flexion and extension half cycles was normalized to % cycle duration. Next, $\dot{\theta }$ was computed from *θ* and normalized to 2*πf*. Likewise, $\ddot{\theta }$ was computed from $\dot{\theta }$ and again normalized to 2*πf*. Hooke’s portraits were constructed separately for flexion and extension half cycles, which were cut from normalized *θ* and $\ddot{\theta }$ time series using time indices of peak flexion and extension. After time-normalization to 100 points per half cycle using a spline interpolation procedure, average *θ* and $\ddot{\theta }$ time series for flexion and extension half cycles were computed for each trial of each participant. A harmonic oscillator produces a straight line in a Hooke’s portrait (i.e., $\ddot{\theta } = - \theta$) and the amount of variance that can be attributed to a harmonic oscillation can be readily quantified by the *r* ^2^ of the linear regression of *θ* onto $\ddot{\theta }$ (i.e., *r* ^2^ = 1 for a purely harmonic oscillation). The explained variance of anharmonicities^4^,^24^,^32^ was expressed as *NL* = 1 − *r* ^2^.

#### Auditory-Motor Coordination

For the acoustic pacing trials, auditory-motor coordination was defined in terms of *ψ*. To compare $\bar{\psi }$ and σ_*ψ*_ between flexion and extension excursion points, *ψ* of unpaced reversal points for single metronome conditions was determined relative to the midpoint between consecutive metronome beeps.^1^

#### EMG

FCR and ECR recordings were band-pass filtered and whitened (see above). To visualize the average muscle activity within a movement cycle, 16 bins were defined in relation to the phase of the hand movement (Θ), defined by tan(Θ) = $\dot{\theta }$/2*πfθ*. Thus, each bin represented an equal part of the phase of the hand oscillation. The first and ninth bin were centered around Θ = 0° (peak flexion) and Θ = 180° (peak extension), respectively. For each bin the RMS value of the EMG was calculated and normalized to MVC.

In addition, a more fine-grained analysis of the bursting behavior of the muscles was performed. Following the method of Staude and Wolf^34^ an approximate generalized likelihood test was used to detect local changes in the statistical properties of the EMG (so-called change times) using sliding test windows *W* of 40 samples and a conservative decision threshold (*h* = 20). RMS values of the EMG between successive change times were calculated to objectively determine which intervals corresponded to bursts. To this end, we first selected the tentative “OFF” intervals with RMS values lower than the median. Subsequently, bursts (and the corresponding onsets and offsets) were identified as those intervals for which the RMS value of EMG activity exceeded the mean plus twice the standard deviation of the EMG activity in these tentative “OFF” intervals. For quantitative analysis of bursting behavior, we determined the number of bursts, the duration of bursts (% cycle duration), normalized EMG activity during a burst (the “ON” amplitude: A_ON_) and normalized EMG activity in between bursts (the “OFF” amplitude: A_OFF_). Finally, to analyze the timing of these bursts, the relative phasing of onsets and offsets with respect to the phase of the movement (Θ_onsets_ and Θ_offsets_, respectively) was determined, with 0° indicating that the onsets/offsets coincided with peak flexion (for ECR) or peak extension (for FCR).

### Statistical Analysis

To determine the effects of acoustic pacing and wrist posture, dependent variables of task performance, local and global kinematics and auditory-motor coordination were submitted to a repeated measures analysis of variance (ANOVA) with within-subject factors direction (2 levels: flexion, extension; for *f*, A_*θ*_, Δ*θ*_req_, and *NL* this factor was redundant), posture (3 levels: flexed, neutral, extended) and pacing (4 levels: no pacing, flex-on-the-beat, extend-on-the-beat, double pacing; because $\bar{\psi }$ and σ_*ψ*_ were not defined for no pacing, three levels were applicable for these measures). Measures related to the analysis of the EMG bursts (i.e., number of bursts, burst duration, A_ON_, A_OFF_, Θ_onsets_ and Θ_offsets_) were initially subjected to a 2 (muscle: FCR, ECR) × 3 (posture) × 4 (pacing) repeated measures ANOVA. However, as explained in the Results section, no meaningful bursting measures could be obtained for FCR in the extended position and ECR in the flexed position. We therefore reduced the factor posture to two levels (i.e., neutral and extreme), where “extreme” corresponded to the flexed wrist posture for FCR and to the extended wrist posture for ECR. Degrees of freedom were adjusted (Huynh–Feldt) if the assumption of sphericity was violated. Effects were labeled significant if *P* < 0.05. *Post hoc* analysis entailed two-tailed paired-samples *t*-tests.

## Results

### Stationary Wrist Posture Trials

**Figure 1:**
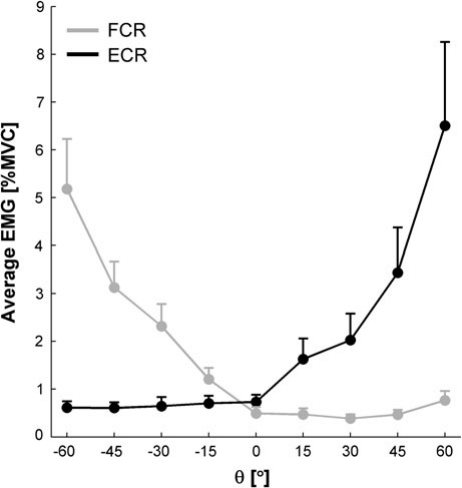
Average EMG for FCR (gray) and ECR (black) muscles as a function of a series of static wrist positions *θ*. Negative *θ* values: flexed wrist posture; positive values: extended wrist postures; 0°: individually determined neutral posture. Error bars represent standard error

In order to validate the experimental manipulation of wrist posture, we first analyzed the muscular activity in the stationary wrist posture trials. As can be appreciated from Fig. 1, which depicts the average EMG of FCR and ECR for static wrist positions *θ*, a passive joint torque indeed needed to be counteracted by activity of the antagonistic muscles in order to maintain a specific *θ* other than neutral (0°). The 2 (muscle) × 9 (wrist positions *θ*) repeated measures ANOVA revealed a significant muscle × wrist position interaction (*F*(1.40,15.43) = 19.1, *P* < 0.001, $\eta_{\text{p}}^{2}$ = 0.634). *Post hoc* analyses indicated that FCR and ECR activity differed significantly from each other for all wrist positions except the neutral position. Muscle activity was statistically symmetrical around 0°, with FCR and ECR activity being similar for each couple of deviations (flexion and extension) from the neutral position.

### Experimental Trials

#### Task Performance

The required amplitude, frequency, and wrist position, indexed by *A*_*θ*_, *f*, and Δ*θ*_req_, respectively, were adequately performed. *A*_*θ*_ was close to the instructed 15° (14.90°±0.21°) and was not affected by wrist posture or pacing. For *f*, a wrist posture × pacing condition interaction was observed (*F*(2.07,22.75) = 5.8, *P* = 0.009, $\eta_{\text{p}}^{2}$ = 0.345), indicating that in unpaced wrist cycling *f* was significantly higher for neutral (1.53 Hz) than flexed (1.50 Hz) or extended (1.49 Hz) wrist postures. With acoustic pacing, no differences between postures were observed; *f* was on average 1.50 Hz with a small standard deviation (0.002 Hz). For Δ*θ*_req_, a near-significant effect of wrist posture (flexed: 0.87°, neutral: 0.23°, extended: −0.99°; *F*(2,22) = 3.4, *P* = 0.052, $\eta_{\text{p}}^{2}$ = 0.235) was observed, suggesting that in extreme wrist positions the center of oscillation deviated towards a more neutral posture. The effect of acoustic pacing was significant (*F*(2.46,27.08) = 5.7, *P* = 0.006, $\eta_{\text{p}}^{2}$ = 0.342), resulting from a shift in center of oscillation towards flexion in flex-on-the-beat (−1.69°) and extension in extend-on-the-beat (1.08°) conditions.

#### Local Kinematics

**Figure 2:**
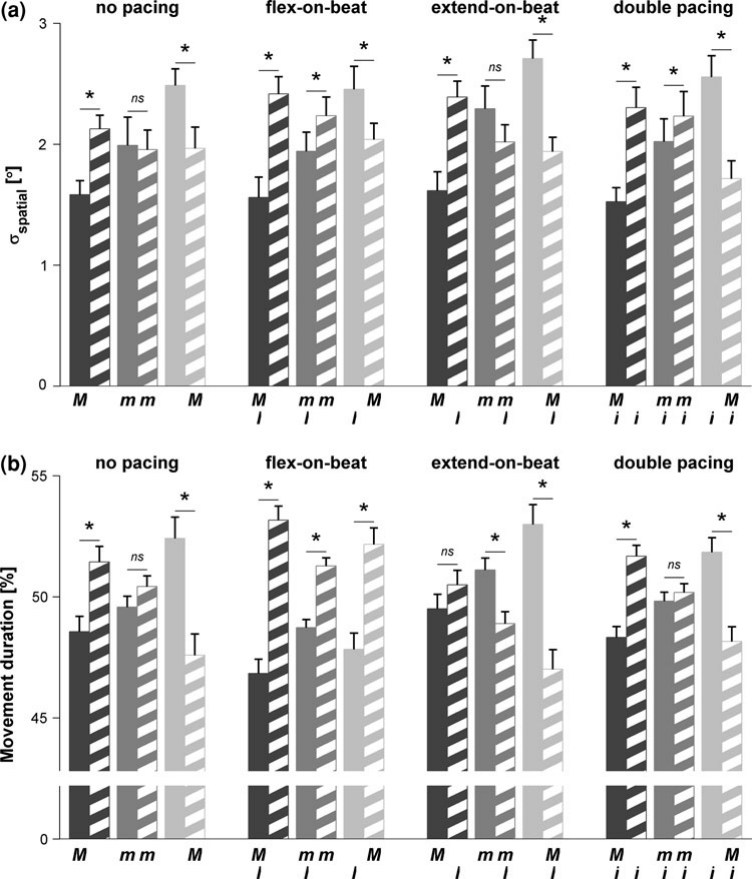
Three-way interaction of (a) spatial reversal point variability (σ_spatial_) and (b) movement duration, presented for all experimental conditions. Flexed, neutral, and extended wrist postures are represented by dark, intermediate, and bright gray bars. Flexion and extension σ_spatial_ or movement duration are indicated by solid and hatched bars, respectively. Asterisks indicate significant differences between both sides within a condition (*P* < 0.05). Error bars represent standard error. Informational timing and neuromuscular mediators are indicated as in Table 1

A significant direction × posture interaction was found for σ_spatial_ (*F*(2,22) = 42.6, *P* < 0.001, $\eta_{\text{p}}^{2}$ = 0.795); flexion σ_spatial_ increased significantly from flexed (1.57°) to neutral (2.06°) to extended (2.55°) postures and vice versa for extension σ_spatial_ (2.31°, 2.11°, and 1.92°, respectively), resulting in significantly lower σ_spatial_ at the reversal point corresponding to the posture manipulation compared to the other reversal point. In the neutral posture no difference in σ_spatial_ was observed between the two reversal points. The direction × posture × pacing interaction was also significant (*F*(6,66) = 3.1, *P* = 0.010, $\eta_{\text{p}}^{2}$ = 0.220; see Fig. 2a). *Post hoc* comparisons indicated that flexion (extension) σ_spatial_ was always lower than extension (flexion) σ_spatial_ with flexed (extended) postures, which was in line with abovementioned direction × posture interaction. In the neutral posture, flexion σ_spatial_ was smaller than extension σ_spatial_ for flex-on-the-beat and double-pacing conditions, suggesting anchoring on peak flexion in these conditions, whereas without acoustic pacing and in the extend-on-the-beat condition no significant differences in reversal point variability were observed.

#### Global Kinematics

A significant direction × posture interaction (*F*(2,22) = 15.0, *P* < 0.001, $\eta_{\text{p}}^{2}$ = 0.577) was found for movement duration. The flexion phase was significantly shorter than the extension phase for the flexed wrist posture (48.31% vs. 51.69%) and vice versa for the extended wrist posture (51.27% vs. 48.73%), whereas they did not differ for the neutral posture (49.81% vs. 50.19%). In addition, the direction × pacing and direction × posture × pacing interactions were significant (*F*(3,33) = 17.4, *P* < 0.001, $\eta_{\text{p}}^{2}$ = 0.613 and *F*(6,66) = 2.8, *P* = 0.017, $\eta_{\text{p}}^{2}$ = 0.203, respectively). The two-way interaction entailed that flexion lasted shorter than extension in flex-on-the-beat conditions (47.8% vs. 52.2%) and vice versa for extend-on-the-beat conditions (51.2% vs. 48.8%); the former difference being more pronounced than the latter (4.4% vs. 2.4%). No difference was obtained for the unpaced (50.2% vs. 49.8%) and double pacing (50.0% vs. 50.0%) conditions. The three-way interaction indicated that the larger difference in the flex-on-the-beat condition was due to the extended wrist posture in which flexion duration (47.8%) did not differ from that in neutral (48.7%) and flexed (46.8%) postures (see Fig. 2b), probably reflecting an attempt to anchor on peak flexion (as evoked by metronome beeps).

**Figure 3:**
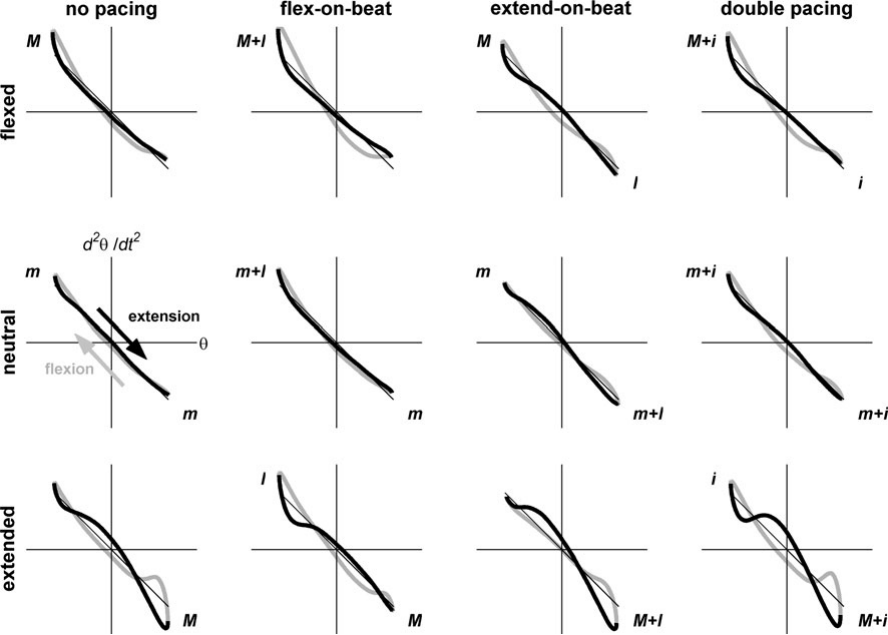
Hooke’s portraits, averaged over participants, as a function of acoustic pacing (columns) and wrist posture (rows) conditions and flexion (gray) and extension (black) half cycles. Informational timing and neuromuscular mediators are indicated as in Table 1

The Hooke’s portraits (Fig. 3) of flexion (gray) and extension (black) half cycles showed systematic deviations from $\ddot{\theta } = - \theta$ (indicating anharmonicities) with extreme wrist positions. This was corroborated by the significant effect of posture (*F*(2,22) = 16.7, *P* < 0.001, $\eta_{\text{p}}^{2}$ = 0.602) for *NL*. Whereas for the neutral posture wrist cycling was quite sinusoidal (0.072), it was significantly less harmonic for flexed (0.099) and extended (0.138) postures, with *NL* being significantly larger for extended than flexed postures. Beyond the observed differences in anharmonicity (i.e., the *NL* value), the presented Hooke’s portraits contained marked signatures of conservative and dissipative nonlinear components. Although we refrained from a formal analysis of these components to keep the study focused, they were most pronounced for the flexed and extended wrist postures. At these postures local stiffness tended to increase towards the anchored reversal point, indicating a so-called hardening spring corresponding to an additional Duffing term in the equation of motion.^4^,^16^,^24^,^32^ This increase in local stiffness, which stands in contrast with the nonlinearly reduced stiffness seen in rhythmic Fitts’ tasks with increasing accuracy demands,^24^ was to be expected given the observed increased agonistic EMG activity required to maintain stationary postures to overcome counteracting passive elastic properties of the “fueled” antagonistic muscles (Fig. 1, see also Guiard^16^). *NL* was not affected by acoustic pacing, but—in line with the three-way interaction for movement duration—the Hooke’s portrait for the extended posture was reversed for the flex-on-the-beat condition (Fig. 3, bottom panels).

#### Auditory-Motor Coordination

Hand movements were on average slightly lagging metronome beats ($\bar{\psi }$ = 27.8°±5.2°). Furthermore, significant direction × posture (*F*(2,22) = 9.8, *P* < 0.001, $\eta_{\text{p}}^{2}$ = 0.470), direction × pacing (*F*(2,22) = 33.2, *P* < 0.001, $\eta_{\text{p}}^{2}$ = 0.751) and direction × posture × pacing (*F*(4,44) = 3.8, *P* = 0.010, $\eta_{\text{p}}^{2}$ = 0.256) interactions were observed for $\bar{\psi }$. These effects basically indicated that $\bar{\psi }$ was smaller in anchored directions. Specifically, *post hoc* analyses indicated that flexion $\bar{\psi }$ was smaller than extension $\bar{\psi }$ (21.9° vs. 28.2°) with a flexed posture and vice versa for an extended posture (29.6° vs. 26.5°); in the neutral posture $\bar{\psi }$ did not differ between sides (30.6° vs. 29.9°). Likewise, in the flex-on-the-beat condition flexion $\bar{\psi }$ was smaller than extension $\bar{\psi }$ (19.4° vs. 26.3°) and vice versa for the extend-on-the-beat condition (30.7° vs. 26.2°); in the double pacing condition $\bar{\psi }$ did not differ between sides (32.0° vs. 32.0°). The three-way interaction reflected the influence of conflicting informational timing and neuromuscular anchoring constraints: flexion and extension $\bar{\psi }$ did not differ in the extend-on-the-beat condition with the wrist flexed (25.2° vs. 26.9°), whereas in the flex-on-the-beat condition with the wrist extended flexion $\bar{\psi }$ was smaller than extension $\bar{\psi }$ (24.2° vs. 32.3°).

For $\sigma_{\psi }$ significant effects of posture (*F*(2,22) = 13.3, *P* < 0.001, $\eta_{\text{p}}^{2}$ = 0.547), pacing (*F*(2,22) = 23.2, *P* < 0.001, $\eta_{\text{p}}^{2}$ = 0.679), and direction × posture interaction (*F*(1.23,13.47) = 6.5, *P* = 0.020, $\eta_{\text{p}}^{2}$ = 0.370) were observed. σ_*ψ*_ was significantly larger with the wrist extended (15.4°) than with flexed (13.6°) or neutral (12.6°) wrist postures. The effect of pacing revealed that σ_*ψ*_ was smaller with double (12.0°) than with single pacing (14.7° and 14.9° for flex-on-the-beat and extend-on-the-beat conditions, respectively). In addition, flexion $\sigma_{\psi }$ was smaller than extension σ_*ψ*_ with the wrist flexed (13.1° vs. 14.2°) and vice versa for the extended posture (16.5° vs. 14.3°); in the neutral posture no difference in σ_*ψ*_ was observed (12.6° vs. 12.6°).

#### EMG

**Figure 4:**
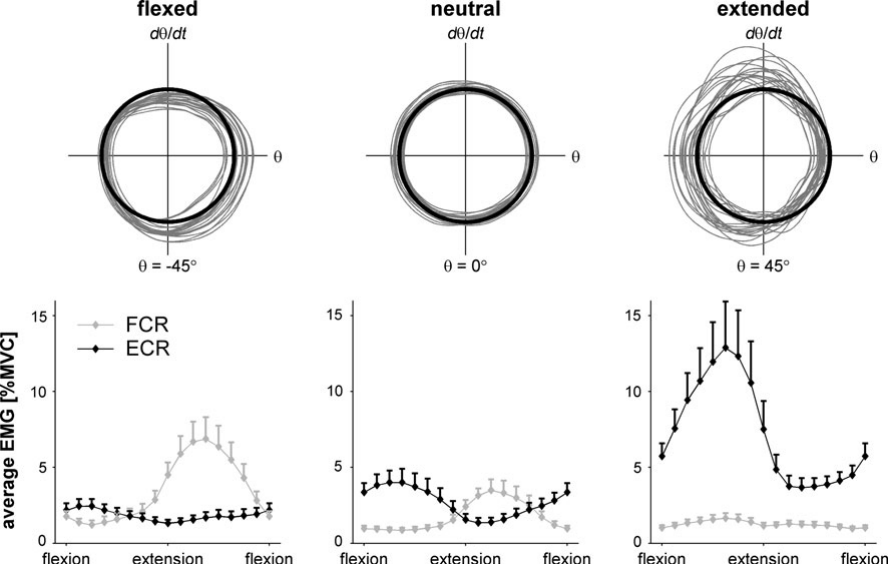
Effects of flexed, neutral, and extended wrist postures on phase portraits and muscle activity. Upper panels: typical phase portraits as obtained for unpaced wrist cycling in the three postures for a single participant. The black circle indicates a harmonic oscillation. Lower panels: for each wrist posture, normalized EMG amplitudes for FCR (gray) and ECR (black) muscles, determined for 16 phases of the movement cycle, were averaged over participants and acoustic pacing conditions; error bars represent the corresponding standard error

In the stationary wrist position trials we had observed systematic increments in EMG amplitudes of ECR in extended positions and FCR in flexed positions (Fig. 1), reflecting a notable passive joint torque. This also had profound effects on the EMG patterns during rhythmic wrist cycling. Figure 4 clearly shows the typical reciprocal FCR-ECR activation pattern for wrist cycling in a neutral posture.^12^,^29^ However, ECR and FCR were less engaged in flexed and extended postures, respectively, indicating that corresponding extension and flexion torques were generated predominantly passively. The representative phase portraits for these unpaced conditions (upper panels) reflect the aforementioned effects of posture on excursion variability (i.e., systematic variations in the locations of local thinning of the phase portrait), movement duration (i.e., asymmetries in peak velocity), and harmonicity (i.e., greater deviation from the dark circle).

The active contribution of each muscle was quantified in terms of the average number of EMG bursts per trial. The significant muscle × posture interaction (*F*(2,22) = 56.4, *P* < 0.001, $\eta_{\text{p}}^{2}$ = 0.837) underscored the general pattern shown in Fig. 4: a smaller number of FCR bursts in the extended (9.8) than in neutral (18.4) or flexed (22.3) postures, whereas for ECR the opposite pattern was observed with less bursts in flexed (9.3) than neutral (16.7) and extended (19.9) postures. In view of the small number of bursts in flexed and extended positions for ECR and FCR, respectively, further analysis of EMG bursts only included flexed and neutral positions for FCR and extended and neutral positions for ECR to preclude potentially unreliable estimates of mean burst duration and mean on- and offset phasing due to inconsistent timing of a small number of bursts only. Furthermore, trials were omitted if less than 10 FCR or ECR bursts were detected, leading to missing values for 3 participants. For the remaining nine participants the analysis focused on neutral and extreme wrist postures, comparing the bursting behavior of FCR in the flexed position to that of ECR in the extended position.

In addition to the global bursting pattern (Fig. 4) and the number of bursts, also EMG amplitudes (A_ON_ and A_OFF_), the burst duration, and on- and offset timing (Θ_onsets_ and Θ_offsets_) were affected (statistics are summarized in Table 2). Only burst duration and on- and offset timing (Θ_onsets_ and Θ_offsets_) were affected by acoustic pacing as well (Table 2). Finally, the absence of significant three-way interactions in EMG activity implied that the posture-induced effects on EMG measures were generally independent from the acoustic pacing effects, motivating separate treatment of these effects in the next sections.

**Table 2:** Statistics of main and interaction effects for EMG measures

Significant (*P* < 0.05) and two near significant effects (*P* = 0.058) are highlighted in dark and bright gray, respectively

#### Effects of Wrist Posture on EMG Measures

Mean burst duration demonstrated significant effects of muscle and posture and the muscle × posture interaction. Bursts were shorter for FCR (31.0%) than for ECR (49.4%) and longer for extreme (43.5%) than neutral (36.9%) postures. FCR bursts were longer in extreme (37.1%) than in neutral (24.9%) positions, whereas such an effect was absent for ECR (49.9% vs. 48.9%, respectively).

For both Θ_onsets_ and Θ_offsets_ a significant effect of muscle was observed. As could be expected, onsets of FCR (−10.5°, just before peak extension) and ECR (−26.3°, just before peak flexion) were approximately a half-cycle apart, with significantly later onsets for FCR. FCR offsets occurred earlier (111.2°, just after peak flexion velocity) than ECR offsets (157.1°, just before maximal extension). The difference in Θ_onsets_ and Θ_offsets_ between muscles accounted for identified differences in burst duration. In addition, for Θ_offsets_ a significant effect of posture was observed, showing later offsets in extreme than in neutral postures (156.6° and 111.8°), probably to overcome increased counteracting passive torque. The significant muscle × posture interaction for Θ_onsets_ indicated earlier onsets for ECR in neutral (−44.6°) than in extreme (−8.1°) positions but, in contrast, earlier onsets for FCR in extreme (−19.3°) than in neutral (−1.7°) postures.

The posture effect and muscle × posture interaction for A_ON_ indicated that A_ON_ was higher in extreme (8.2%) than neutral (5.9%) positions and that this amplitude difference was larger for ECR (9.7% vs. 5.9%) than for FCR (6.6% vs. 5.8%). For A_OFF_ significant muscle, posture, and muscle × posture interaction effects were obtained. A_OFF_ was higher for ECR (2.4%) than for FCR (1.4%) and higher in extreme (2.2%) than neutral postures (1.6%). The interaction indicated that both main effects were due to larger A_OFF_ for ECR in extreme (3.0%) than neutral (1.7%) postures, as no other significant differences were observed (A_OFF_ FCR: 1.4% vs. 1.4%).

#### Effects of Acoustic Pacing on EMG Measures

For burst duration a significant muscle × pacing interaction was observed, indicating that ECR bursts lasted relatively shorter (44.3%) in the extend-on-the-beat condition than in other pacing conditions (unpaced: 48.5%, flex-on-the-beat: 53.7%, double pacing: 51.1%). In contrast, FCR bursts lasted relatively longer in the extend-on-the-beat condition (34.3%) than in unpaced (29.5%), flex-on-the-beat (29.4%), and double pacing (30.8%) conditions. For Θ_onsets_ a near significant muscle × pacing interaction was observed, which might suggest that shorter burst durations in anchored directions were (in part) due to later onsets of corresponding EMG activity (FCR; unpaced: −9.1°, flex-on-the-beat: −7.0°, extend-on-the-beat: −19.0°, double pacing: −7.0°, and ECR; unpaced: −30.4°, flex-on-the-beat: −35.7°, extend-on-the-beat: −14.9°, double pacing: −24.4°). This suggestion was underscored further by the fact that Θ_offsets_ was affected significantly by pacing and the muscle × pacing interaction. FCR offsets occurred earlier in pacing conditions for which a shorter burst duration was observed (unpaced: 100.5°, flex-on-the-beat: 106.9°, double pacing: 110.8°) than for the extend-on-the-beat condition (Θ_offsets_ = 126.6°). Likewise, Θ_offsets_ for ECR occurred somewhat earlier in the extend-on-the-beat condition (152.6°) than in other pacing conditions (unpaced: 156.1°, flex-on-the-beat: 156.5°, double pacing: 163.4°).

## Discussion

The present study examined the location and degree of anchoring in continuous rhythmic movements by systematically manipulating informational and neuromuscular constraints on anchoring (see Table 1). Marked anchoring effects were observed when balanced informational timing or neuromuscular anchoring constraints (*i* or *m*) were combined with either directional informational timing or directional neuromuscular constraints on anchoring (*I* or *M*). Furthermore, conditions with conflicting *I* and *M* were instrumental in delineating the relative contribution of the two types of constraints on task execution. In general, temporal and relative phasing aspects of anchoring appeared to depend on informational timing constraints, even for flexed and extended wrist postures. In contrast, the neuromuscular constraints were found to have a particularly strong impact on spatial aspects of anchoring (as indexed by σ_spatial_), regardless of pacing condition. Ambiguous kinematic results for conflicting *I* and *M* conditions were resolved by complementary EMG findings, which revealed distinct neuromuscular signatures of anchoring and differences therein between flexor and extensor muscles. Below we discuss the theoretical implications of these findings for understanding how the brain controls rhythmic hand movements. To facilitate the line of reasoning, we first focus on the findings with regard to neuromuscular constraints and then on the informational timing constraints.

### Neuromuscular Constraints on Anchoring

As expected, wrist cycling with flexed and extended wrist postures mediated anchoring at peak flexion and peak extension, respectively, as evidenced by reduced variability at the anchored reversal point, shorter movement duration in the anchored direction and decreased overall harmonicity (Figs. 2–4). The suggestion that at these anchor points task-specific neuromuscular properties related to energy storage and release^16^,^32^ are exploited, was supported by EMG results showing counteracting passive moments in static (Fig. 1) and dynamic (Fig. 4) situations. Notably, the amount of muscular activity scaled with deviations from neutral static wrist positions, while the phase-dependent reciprocal bursting activity typically seen for rhythmic wrist cycling in a neutral posture^12^,^29^,^37^ disappeared with extreme wrist postures (i.e., ECR was predominantly active in wrist cycling with an extended posture and FCR for oscillations with the wrist flexed).

We also observed marked differences between flexor and extensor muscles in terms of burst duration (FCR bursts lasted shorter), timing (FCR onsets were later, offsets earlier) and amplitude (FCR amplitude was lower), which, in all likelihood, are related to neuromuscular differences between flexors and extensors.^2^,^11^,^13^,^15^ Increased ECR amplitudes during and in between bursts may have accounted for the reduced harmonicity of wrist oscillations in extended postures (Fig. 3). More interestingly, the observed significant muscle × posture interaction for nearly all EMG measures (Table 2) revealed distinct adaptations of FCR and ECR to changes in wrist posture. Higher amplitudes and longer burst durations were to be expected^37^ in extreme positions to compensate for increased counteracting passive moments. However, our findings were more subtle in that ECR modulated merely its activity level, whereas FCR modulated its duration and timing. The ability to utilize variations in timing, burst duration, and muscular activity during and in between bursts may be critically important for achieving dexterity, efficiency, and flexibility in motor control. Apparently, flexor muscles are better suited for this purpose than extensor muscles^2^,^15^ because the increased activity level observed for extensor muscles reflects a less economical adaptation to changes in wrist posture.

### Informational Timing Constraints on Anchoring

Single metronome conditions induced differential anchoring phenomena in expected directions: in the flex-on-the-beat condition, lower flexion than extension σ_spatial_ was observed and vice versa for the extend-on-the-beat condition (Fig. 2). In addition, shorter movement durations and smaller deviations in the relative phasing between hand excursions and metronome beats $\bar{\psi }$ were observed in the anchored directions. These effects were evident for wrist cycling in a neutral posture and in extreme postures with coinciding informational timing and neuromuscular constraints on anchoring (*I* and *M*). For all these conditions the measures progressed in similar directions, lending credibility for the assumption that they reflect local (σ_spatial_ and $\bar{\psi }$) and global (movement duration) aspects of anchoring.^32^

In general, the effects of the informational timing and neuromuscular constraints on anchoring were largely independent, with the exception of some three-way interactions that revealed subtle task-specific trade-offs between the two types of constraints. These interactions were all associated with flex-on-the-beat with extended wrist and extend-on-the-beat with flexed wrist conditions. In the former condition extension reversal variability was lower, indicating anchoring at peak extension, while flexion movement duration was shorter and flexion $\bar{\psi }$ was smaller, indicating additional anchoring at peak flexion. Such opposite effects were not observed for the extend-on-the-beat condition with the wrist flexed. The strength of the induced informational timing constraint on anchoring at peak flexion for these two conditions with conflicting *I* and *M* was underscored further by Hooke’s portraits (Fig. 3), providing a qualitative image of the conservative and dissipative nonlinear components giving rise to the observed anharmonicity.^4^,^24^ Whereas for the extend-on-the-beat condition with the wrist flexed the contribution of the nonlinear components in question were qualitatively similar to the other three conditions with a flexed wrist, this was not the case for the flex-on-the-beat condition with the wrist extended, which was a more pronounced mirror image of the observed anharmonicity for the other three pacing conditions with the wrist extended, strongly suggesting anchoring to peak flexion. In combination, these results suggest that participants were less successful in anchoring peak extension to the beat with the wrist flexed than anchoring peak flexion to the beat with the wrist extended, possibly due to superior dexterity of flexor muscles over extensor muscles and associated lower attentional demands.^8^ Indeed, we also observed a bias towards anchoring on peak flexion with double pacing in a neutral posture, albeit only in terms of reduced reversal point variability (Fig. 2a).

Interestingly, changes in muscle EMG induced by informational timing constraints differed from those induced by neuromuscular constraints: bursts were generally shorter in anchored directions with somewhat delayed onsets and earlier offsets of muscular activity. As Wachholder and Altenburger^37^ reported similar EMG anchoring characteristics, it is likely that both types of constraints played a role in that study as well. Specifically, the longer pause between offsets of accentuated and onsets of unaccentuated movement phases^37^ resembled our EMG findings regarding informational timing constraints on anchoring, whereas the longer periods of increased muscular activity^37^ were congruent with our EMG findings regarding neuromuscular constraints on anchoring (i.e., FCR showed longer bursts while ECR showed increased A_ON_). Finally, for conditions with conflicting *I* and *M* the complementary EMG findings were instrumental in delineating the relative contribution of the two types of constraints on task execution, uncovering distinct posture-dependent neuromuscular signatures of anchoring and differences therein between flexor and extensor muscles.

### Coda

In the present study we employed motor variability as a window into motor control, to uncover how the central nervous system exploits informational timing (*viz*. acoustic pacing) and neuromuscular (*viz*. wrist orientation) properties in the control of rhythmic movements. We found that the anchor-based discretization of the control of continuous rhythmic wrist movements is determined by both types of properties in a task-specific manner with subtle interactions between the two. This implies that the central nervous system organizes the timing of reversal points at metronome beeps, especially if it can take advantage of neuromuscular properties at those points. In so doing, we illustrated exactly how movement variability may serve as a window into motor control. Specifically, the scouting of local thinning of the phase portrait (i.e., reduced reversal point variability) facilitated the identification of the anchor point(s) in the movement cycle, independent of the presence of acoustic pacing stimuli. With acoustic pacing, the reduced relative phase variability between reversals and metronome beeps pointed at anchoring as well, which often but not always coincided with the local thinning of the phase portrait. By complementing these kinematic signs of anchoring with analyses of the underlying EMG activity, we linked the concept of anchoring (typically based on kinematic features) to the realm of neurophysiology (with inferences based on EMG characteristics), thereby providing a complementary entry point for understanding how the brain controls rhythmic hand movements. Collectively, our findings suggest that the number and precise location of anchor points may be (co-)determined by prevailing cost functions related to task performance (more anchor points may be beneficial; *viz*. lower σ_*ψ*_ for double than single metronome conditions^14^,^21^) and task economy or computational burden (less anchor points may be more economical^31^).

Inspired by Bernstein,^5^ proponents of the dynamical systems approach have asserted that coordinated movement is the outcome of a confluence of organismic, environmental, and task constraints.^10^,^25^ However, in order to substantiate such assertions, the constraints in question need to be identified and their convergence needs to be unpacked. The experimental study and analysis of motor variability and anchoring along the lines pursued in the present study may help to achieve this goal, as it serves to identify such constraints in the form of neuromuscular and informational timing mediators of anchoring, and how they are combined in the instantiation of specific forms of coordination, given the prevailing task demands associated with the stability, accuracy, and efficiency of performance.
